# Role of digital pathology in diagnostic histopathology in the response to COVID-19: results from a survey of experience in a UK tertiary referral hospital

**DOI:** 10.1136/jclinpath-2020-206786

**Published:** 2020-07-02

**Authors:** Lisa Browning, Eve Fryer, Derek Roskell, Kieron White, Richard Colling, Jens Rittscher, Clare Verrill

**Affiliations:** 1 Department of Cellular Pathology, Oxford University Hospitals NHS Foundation Trust, Oxford, Oxfordshire, UK; 2 NIHR Oxford Biomedical Research Centre, Oxford, Oxfordshire, UK; 3 Nuffield Department of Surgical Sciences, University of Oxford, Oxford, Oxfordshire, UK; 4 Department of Engineering Science, University of Oxford, Oxford, Oxfordshire, UK

**Keywords:** education, information technology, pathology, surgical, hospital, pathology department

## Abstract

The COVID-19 pandemic has challenged our diagnostic services at a time when many histopathology departments already faced a diminishing workforce and increasing workload. Digital pathology (DP) has been hailed as a potential solution to at least some of the challenges faced. We present a survey of pathologists within a UK National Health Service cellular pathology department with access to DP, in which we ascertain the role of DP in clinical services during this current pandemic and explore challenges encountered. This survey indicates an increase in uptake of diagnostic DP during this period, with increased remote access. Half of respondents agreed that DP had facilitated maintenance of diagnostic practice. While challenges have been encountered, these are remediable, and none have impacted on the uptake of DP during this period. We conclude that in our institution, DP has demonstrated current and future potential to increase resilience in diagnostic practice and have highlighted some of the challenges that need to be considered.

## Introduction

The current pandemic has placed the National Health Service (NHS) into a position where the workforce has needed to adapt rapidly to a variety of challenges, one of which being the recommendation for remote working where possible. The majority of us have quickly become accustomed to the utility of technology such as videoconferencing during this crisis, and other technologies are opening doors to the opportunity for remote working, protecting the workforce during these challenging times while maintaining clinical services and other aspects of our work such as training and management.^1^

Histopathology presents its own specific challenges within this context, partly because there are aspects of the job that simply cannot be done remotely, for example, the handling of surgical and cytology specimens. Reporting of clinical cases on glass slides is possible remotely, necessitating the transportation of the cases, and this is familiar to many histopathologists who have worked flexibly prior to the current crisis. However, the advent of digital pathology (DP) and the expansion of this facility within an increasing number of cellular pathology departments in the UK and across the world offers opportunity for truly remote diagnostic reporting, negating the requirement for transportation of cases and offering a potential solution to unpredictable workforce crises through the sharing of workloads within departments and even between departments that are geographically separate.

In addition to DP as a potential solution for current and future workforce challenges,^2–4^ increasingly recognised are the wider benefits of a digital transformation for diagnostic collaboration and multidisciplinary working to improve patient care,^2 5^ for education and training purposes,^6–8^ clinical trial working and research,^9^ and for the future application of artificial intelligence and the promise of computer-aided or computer-augmented diagnosis.^10–12^

For the purpose of primary diagnostic reporting, there are stringent guidelines on training in reporting digitally in order to maintain clinical safety.^13^ With the potential utility of DP to enable continuity of clinical services during this period of crisis, but in recognition of the need for guidance specifically on the remote use of DP, including among pathologists not yet fully ‘validated’, the Digital Pathology Committee of the Royal College of Pathologists responded by issuing ‘Guidance on remote reporting of DP slides during periods of exceptional service pressure’,^14^ which was issued in parallel with ‘Best practice recommendations on reporting cellular pathology samples at home’.^15^ This guidance provides pathologists with an overview of risk associated with remote/home digital reporting and with a risk mitigation strategy. It is recognised within these guidance documents that DP-related activities such as review for auxiliary test requests and secondary review/multidisciplinary team (MDT) review are associated with a different level of risk to primary diagnostic reporting. Guidance has also been issued by the College of American Pathologists (CAP); ‘Remote sign-out of cases with digital pathology FAQs’,^16^ in response to the efforts of professional bodies, including the CAP, in gaining approval from the Centers for Medicare and Medicaid Services to allow pathologists to review slides and sign out cases at a remote (unlicenced) location.

In our centre, we have the capability to operate a fully digital diagnostic service for surgical pathology within in the setting of the NHS. We have undertaken a graduated roll out of DP across specialties within our tertiary referral centre, with all teams having started or completed validation for DP reporting. We have seen the uptake of DP across the department increase during this current crisis, both in terms of actual numbers of pathologists using the DP platform for aspects of diagnostic work and in the breadth of utility of DP. We wished to capture a snapshot of the impact of COVID-19 on the use of DP and to potentially identify utilities of DP that could be expanded on through sharing of experiences and areas for which additional support might be of benefit.

## Methods

A survey was circulated via the online SurveyMonkey survey tool (www.surveymonkey.co.uk) to 34 independently reporting histopathologists within our tertiary referral hospital (online supplementary table). The aim of the survey was to gather opinions with regard to the use of DP within the clinical setting and to assess how this may have been impacted on by the COVID-19 pandemic. The survey results would potentially inform improvements during the upscaling of this facility for those pathologists accessing DP both remotely and within the workplace, as well as providing a better understanding of potentially under-recognised challenges that might impact more generally as pathologists adopt DP more widely within the setting of the NHS.

10.1136/jclinpath-2020-206786.supp1 Supplementary data

## Results

Eighteen pathologists responded to the survey (53% response rate), and all answered in full.

**Figure 1:**
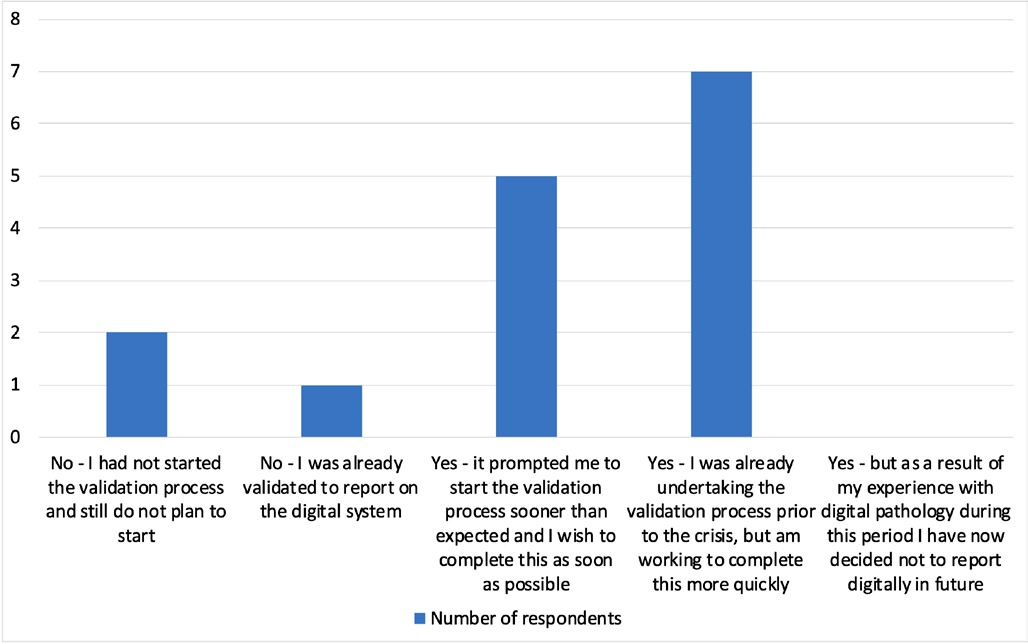
Has the current crisis and the need to consider remote working impacted on your uptake of the digital pathology validation process?

Overall of those who responded, 14/18 are currently using the digital platform, compared with 9/18 prior to the crisis, and 12/18 had either newly started the validation process or were aiming to complete this more rapidly than previously (figure 1).

Results indicated that the digital platform is being used in a variety of ways; 9/18 reported using the digital platform for reporting of some (6/18) or all (3/18) clinical cases, although the application of the term ‘reporting’ was not specific and encompassed digital viewing of whole slide images (WSI) within the diagnostic process but not limited to authorisation of a case based on the review of WSI. For example, 15/18 reported using DP for a ‘quick review of a case to determine whether levels/immunohistochemistry/special stains were needed’, and 12/18 reported using DP for second opinions (within the Trust). At the current time, there is no established and robust facility to allow sharing of cases digitally outside the Trust or local network, and this was commented on by several respondents. This applies particularly to those working within very specialised areas for whom sharing cases digitally with other centres would facilitate the provision of remote diagnostic services or facilitate the sharing of challenging cases with other centres with expertise. Similarly, it was highlighted that the majority of cases referred into the department for MDT meeting discussion or specialist opinion are not currently part of the routine digital workflow that are otherwise all scanned routinely.

Considering where people use the digital platform (in the clinical diagnostic setting), 9/18 reported using it in the workplace and at home, with 5/18 only using it at work and 2/18 only using it at home. This was reported to be a change for almost half of the respondents, with 8/18 reporting that as a result of COVID-19, they have now started accessing DP from home or are using it from home more frequently than before.

### Impact of access to the DP system during COVID-19

Respondents were asked specifically about the impact of access to the DP system during the current crisis in relation to various aspects of clinical work. Nine out of 18 agreed that DP had facilitated maintenance of their diagnostic practice while remote working, with 6/18 and 7/18 respectively agreeing that DP had eased workforce crises during this period and reduced potential impact on turnaround times, thus directly benefitting patients. There was an overall positive response regarding the implementation of DP, with 14/18 agreeing that DP is a positive step for their specialty team and 16/18 agreeing that they would likely continue reporting digitally beyond this crisis; no respondents stated that they would not report digitally in the future as a result of their DP experience to date.

DP has also played a positive role in MDT working. Ten out of 18 reported that they have demonstrated digital images in an MDT meeting setting, including remotely via secure screen share during videoconferencing. Furthermore, access to DP permits remote preparation/case review prior to an MDT, and 14/18 of respondents reported using DP for this purpose, six of them newly adopting this practice during the pandemic.

Examples were sought (within free-text comments) as to how DP had provided a solution to specific issues during this period (table 1), although in fact it can be seen that most will be valid as benefits of DP beyond this crisis.

**Table 1:** Examples given of specific instances of where digital pathology has provided a solution to specific issues during this period or how it is anticipated to do so in the future

Workflow related	Facilitating double reporting and second opinions from colleagues, including from those who are working at home (11 comments). Double reporting (at work) without need to use multiheaded microscope (one comment). Quick triage of case to assess need for immunohistochemistry, improving timeliness of reporting of the case (two comments), for example, triage remotely at home prior to being in the department to review the glass slides. Reporting (remotely) of urgent cases (one comment). Full reporting of cases from home (one comment). Ease of access to previous histology, even from home (one comment). Taking snapshot images of a case to share remotely prior to formal (external) referral (one comment).
Multidisciplinary team meeting (MDT) related	Provision of a solution for remote MDT working, showing cases remotely (or in future live) at an MDT and reviewing cases prior to an MDT (seven comments).
Teaching/training related	Sharing of cases with trainees for training/educational purposes (two comments).
Workforce health and safety related (specific to COVID-19)	Reducing potential (risk of infection) from handling of slides and slide trays between colleagues for a second opinion.
Workforce health and safety related (general)	Reduction in back and neck pain associated with use of the microscope.
Governance related	Potentially negating the need to remove glass slides from the department for home working (one comment).

Examples were also sought (as free-text comments) of specific challenges faced during the upscaling of DP (table 2). Again, some of those raised are general issues unrelated to the current crisis. Many of the challenges are related to the ‘set-up’ for remote working, including difficulties with internet speed and workstations. More than one respondent reported personal investment in upgrading internet access, a new computer and/or a suitable screen, and it was commented that this reflected inequality across medical specialties in terms of corporate investment into digital technologies to facilitate home working. None of the challenges raised appear however to have impacted on the uptake of DP.

**Table 2:** Issues reported to have impacted on the use of the digital pathology system/information management system (IMS)

Internet speed and/or loss of connectivity to the IMS	Seven comments (all related to home use). For example, ’25 Mbit bandwidth at home not enough to avoid delays & pixelation when viewing slides’. ‘slow internet bandwidth speed at home limits usability’. ‘… not sure how much of this is due to the VPN being the limiting step and how much general bandwidth’. ‘on one occasion I had to give up on a case as the slides were loading so slowly’. ‘occasional loss of connection to the IMS, particularly from home’. ‘Bandwidth is the main and a major problem’.
Unsuitable quality of screen (at home)	Two comments.
Need to upgrade computer (at home)	One comment.
Mouse to manipulate images	Two comments For example, ‘(would be) useful to have something other than a standard mouse’.
Occasional out of focus slides	Seven comments.
Digital workflow incomplete	One comment For example, ‘Request forms not on IMS means that (it is) difficult to sign cases out digitally’.

Another recurrent theme was the importance of absolute clarity in the guidance pertaining to the utility of DP in diagnostic practice, especially with respect to remote working. Three-quarters of the respondents were aware of the recent RCPath guidance on remote reporting of DP slides,^14^ which was written in direct response to the current pandemic; however, respondents indicated the desire for specific additional local guidance on this issue.

## Discussion

DP provides an opportunity for pathologists to view digital images of glass slides on a workstation that can be remote from their usual place of work, for example, at home. In our experience as a department in transition to DP, the utility of such a set-up is broad and not limited to the full sign out of clinical cases, providing additional workflow solutions even for those who are not fully validated to digitally report a case,^13 14^ such as the remote provision of a second opinion, access to cases for simple triage for additional work such as immunohistochemistry and access to previous histology. Indeed, three-quarters of those surveyed indicated that they had made use of remote DP for triage of cases and two-thirds used DP for second opinions. This was recognised to be timesaving when it was not possible to be physically in the workplace for a period of time, and half felt that remote access to DP had facilitated their diagnostic practice, particularly around the access to colleagues for second opinions/double reporting. Specifically, during this current crisis, DP also reduces the potential infection risk of ‘contact’ with glass slides for those working on or off site, and with pathologists able to work with reduced interpersonal contact, this adds to workforce resilience. Even within the workplace, it was noted that by replacing double-heading at the microscope, DP allows safe distance between colleagues to be maintained while discussing a case.

Within a centre in the advantageous position of having access to a digital workflow, while the transition to digital diagnostic reporting was already underway, the COVID-19 crisis has expedited the process of transition in order that access to DP be made available across specialty teams, including those not reporting digitally prior to the crisis. We have seen a 25% increase in uptake of DP during this period, with pathologists keen to fully validate digitally as a result of the crisis, and it has been possible to provide remote training and ongoing support for this transition successfully via videoconferencing. The survey of our pathologists indicates that DP has been seen as a positive step forward, providing a means during this unprecedented period to facilitate continuity of service provision, and in an inclusive way with access to cases available digitally to all of those working remotely. This has potentially lessened the impact of both short-term and longer term workforce challenges. Those working remotely need an appropriate set-up, including secure access to the departmental laboratory information system, to the request forms (especially if authorising the case remotely) and a means to access any additional clinical details for the patient^14^; however, this is not specific to digital reporting, and in our experience, such a set-up was already in place for those pathologists who had worked from home prior to the crisis and was therefore easy to roll-out to those who did not have such access. The request forms are now scanned and accessible within the digital platform, a change that has been made in direct response to our survey in order to improve workflow. MDT working has also benefitted, with three-quarters of those surveyed using DP as a means to review cases remotely during MDT preparation and with half of respondents using it for live demonstration of slides during remote MDTs conducted via secure videoconferencing facilities, thus benefiting the clinical discussion and also providing the pathologist with a platform to maintain their ‘role’.

The work of the RCPath and CAP emphasises the importance of a timely and cohesive response from our professional bodies to support flexibility in working practice in order to maintain quality clinical services while protecting an already challenged workforce. However, in our experience, and as evident from our survey, it is important to have timely specific local guidance for pathologists on this matter in which risk assessment and risk mitigation strategies and any specific local expectations in this regard are clarified, and we have developed additional guidance locally in response to this survey. Provision of such guidance may be a challenge for those institutions who are early adopters of the technology, and sharing of good practice guidance in this regard may therefore be beneficial to the wider community going forward.

The maintenance of education and training is another important application of DP, brought to the forefront at a time when training opportunities may be reduced with consultant pathologists working off-site and when trainees may be isolated at home or otherwise redeployed to the ‘front line’ of clinical service provision. Other recent authors^17–19^ specifically identify DP as a training tool for pathology residents during this period as it is a portal for access to clinical cases. While not specifically explored within our survey, we have made use of the digital platform to share live clinical cases remotely with our trainees, either via screen share during secure videoconferenced discussions with trainees or by simply sharing an email link to a digital case with associated email interaction. This has been beneficial for trainees on ‘attachment’ who cannot sit beside a consultant histopathologist at a multiheaded microscope, and it has provided an opportunity for group teaching sessions with trainees, potentially even across geographical sites. Such teaching sessions have been a means to reach out to those isolated at home or working temporarily outside the specialty during this crisis, as well as facilitating the opportunity (which extends beyond this crisis) for those who are out of training, for example, during a period of research, parental or other leave, to maintain both contact and knowledge/skills and potentially lessen the impact of time away from clinical practice. This has been well received by the trainees who have indicated a keenness to continue with these teaching methods going forward. The crisis has also provided an opportunity for trainees to have increased exposure to DP per se, beyond that which they might have done in prepandemic times with more traditional working patterns.

There are of course challenges that need to be considered during the implementation of DP, and these are well documented elsewhere.^5^ However, this acute period serves to highlight those issues with real and immediate impact on the utility of DP. In our experience, those most frequently reported were technical issues such as internet speed and access to an appropriate workstation, workflow considerations including a pipeline for the identification of out of focus cases for rescanning, as well as the need for clear and workable guidance to the workforce on transitioning to digital reporting and on remote reporting. None of these issues are insurmountable, and this period of unprecedented challenge has reinforced the value and importance of the hard work and commitment that has been shown in undertaking this digital transformation and of its current and future value in ensuring quality and sustainability of our clinical services.
