# Supplementary material for: Role of digital pathology in diagnostic histopathology in the response to COVID-19: results from a survey of experience in a UK tertiary referral hospital
Source: J Clin Pathol. 2020 Jul 2;74(2):129–32. doi: 10.1136/jclinpath-2020-206786 (PMC7841475; doi:10.1136/jclinpath-2020-206786)
Supplement: Supplementary data [file jclinpath-2020-206786supp001.pdf]

Q1. How would you best describe your current level of experience as a histopathologist (please *tick one* of the following options)?

I am a histopathologist with > 20 years experience (including training)

I am a histopathologist with 15-20 years experience (including training)

I am a histopathologist with 10-15 years experience (including training)

I am a histopathologist with 5-10 years experience (including training)

Q2. Prior to the COVID-19 crisis and the need to consider remote working, were you using the digital pathology system (IMS) in your clinical diagnostic practice?

IMS = Information Management System

No - and I was not planning to start the validation process

No - although I had planned to start the validation process

Yes, but only occasionally and I was not reporting all clinical cases digitally

Yes, on a daily basis but I was not reporting all clinical cases digitally

Yes, and I was already reporting all clinical cases on the digital system (where possible)

Other, please specify:

Q3. Has there been a change in your use of the digital pathology system (IMS) in your clinical diagnostic practice during this period of crisis?

No

Yes, I have now started using the digital pathology system (IMS) for diagnostic work, which I was not doing previously

Yes, although I had experience using the digital pathology system (IMS) for diagnostic work previously, I am now using the system more regularly

Other, please specify:

Q4. Prior to the COVID-19 crisis and the need to consider remote working, how would you describe your progress with the digital pathology validation process (please tick one of the following options)?

I had not started the validation process and did not plan to start

I had not started the validation process but was planning to start

I was in phase 1 of the validation process

I was in phase 2 of the validation process

I was already validated to report on the digital system

I had started the validation process but was unsure whether I wanted to complete it, i.e. I was not sure that I wanted to report digitally going forward

Other, please specify:

Q5. Has the current crisis and the need to consider remote working impacted on your uptake of the digital pathology validation process (please tick one of the following options)?

No - I had not started the validation process and still do not plan to start

No - I was already validated to report on the digital system

Yes - it prompted me to start the validation process sooner than expected and I wish to complete this as soon as possible

Yes - I was already undertaking the validation process prior to the crisis, but am working to complete this more quickly

Yes - but as a result of my experience with digital pathology during this period I have now decided not to report digitally in future

Other, please specify:

Q6. Are you currently using the digital pathology system (IMS) in your clinical diagnostic practice?

No

Yes, but only occasionally and I am not reporting all clinical cases digitally

Yes, on a daily basis but I am not reporting all clinical cases digitally

Yes, and I am reporting all clinical cases on the digital system (where possible)

Yes - but I am only using this for purposes other than actual reporting of a clinical case, for example to assess requirement for immunohistochemistry/levels/further opinion

Other, please specify:

Q7. If you are using the digital pathology system (IMS) in your clinical diagnostic practice, have you encountered any issues that have impacted on this, for example bandwidth speed, out of focus slides etc. Please comment.

Q8. Regarding all potential uses of digital pathology on the IMS, which of the following purposes are you currently using it for (please tick all that apply);

For diagnostic work (this will be elaborated on in the next question)

For discussion of cases at MDTs (showing digital slides from the IMS on screen share)

For training/educational purposes

Other, please specify:

Q9. Regarding all potential uses of digital pathology in the clinical diagnostic setting on the IMS, which of the following purposes are you currently using it for (please tick all that apply);

For full reporting of all clinical cases

For full reporting of some clinical cases

For quick review of clinical cases to determine whether they need levels/immuno/special stains

For giving/receiving second opinions to/from colleagues at OUH

For giving/receiving second opinions to/from colleagues outside OUH (for example by taking images of clinical cases that can be shared anonymously)

Other, please specify:

Q10. Regarding your current use of digital pathology in the clinical diagnostic setting, where are you using the system;

At home only

At work only (at OUH)

Both at work and at home

Other, please specify:

Q11. Regarding your use of digital pathology currently in the clinical diagnostic setting compared with that prior to the crisis, has there been a change in where you are using the system;

No - I have not been using digital pathology in a clinical diagnostic setting prior to the crisis or since

No - I was previously accessing the digital pathology system (IMS) at work and at home, and this has not changed

Yes - although I previously accessed the digital pathology system at work and at home, I am now accessing it from home more frequently

Yes - I was previously only accessing the digital pathology system (IMS) at work but I am now using it at home as well

Other, please specify:

Q12. Considering the impact of access to the digital pathology system (IMS) during this time of crisis, please answer the following;

	STRONGLY DISAGREE	DISAGREE	NEUTRAL	AGREE	STRONGLY AGREE	UNSURE	N/A
Access to digital pathology for reporting of clinical cases has facilitated the maintenance of my own diagnostic practice whilst remote working during this crisis							
I feel satisfied with the quality of clinical work that I am able to achieve using the digital pathology system							
Access to digital pathology has facilitated double							

reporting of cases within our team during this crisis							
Access to digital pathology has eased workforce issues within our team during this crisis							
Access to digital pathology has reduced the potential impact of remote working on turnaround times in our team during this crisis							
Access to digital pathology for reporting of clinical cases has been a positive step forward for our team							
I feel more confident in using the digital reporting platform than I did prior to the crisis							

The expedited transition process to aide remote working has been a positive step for me as I may not otherwise have taken the step toward digital reporting							
Having access to digital pathology for reporting of clinical cases is a better option than having a potentially lesser quality microscope at home for reporting							
I am likely to continue to report on the digital system after the crisis							

Q13. Please can you provide examples of specific instances of where digital pathology has provided a solution to specific issues you/your team have faced (e.g. diagnostic or workforce related), or how it is anticipated to do so in the future for you/your team? An example might be facilitating double reporting of cases where there this is otherwise not possible with remote working;

Q14. Regarding the utility of access to digital pathology slides on the IMS for the purposes of your MDT preparation, please indicate which of the following best describes your practice;

I have not utilised the digital pathology system (IMS) for the purpose of MDT preparation to date

I had been utilising the digital pathology system (IMS) for the purpose of MDT preparation prior to the crisis, and my practice in this respect has not changed

I had been utilising the digital pathology system (IMS) for the purpose of MDT preparation prior to the crisis, however I am now doing this from home and from work (previously just at work)

I have now started utilising the digital pathology system (IMS) for the purpose of MDT preparation having not done this previously

Other, please specify:

Q15. Regarding the sharing of digital pathology slides within the MDT setting, please answer the following (please tick all that apply);

I have not demonstrated digital pathology slides within the MDT setting to date

I was demonstrating digital pathology images on the IMS in the MDT setting prior to the crisis

I have been demonstrating digital pathology images on the IMS using screen sharing during remote MDT meetings during this period of remote working

I would like to be able to demonstrate digital pathology images on the IMS using screen sharing during remote MDT meetings , but do not know how to do this

I plan to/would like to continue to demonstrate digital pathology images on the IMS during MDT meetings in future (beyond this crisis period)

I have found it easy to share the digital cases on the IMS remotely via screen sharing

Sharing of the digital cases on the IMS via screen sharing has been beneficial to the MDT discussion

I have received positive feedback from the clinical team with regard to the sharing of digital cases during remote MDTs

Comment;

Q16. I am aware of the RCPath 'Guidance on remote reporting of digital pathology slides during periods of exceptional service pressure';

No

Yes

Q17. Do you have any further comments to add?
